# Supplementary material for: An Ultra-Sensitive Technique: Using Pv-mtCOX1 qPCR to Detect Early Recurrences of Plasmodium vivax in Patients in the Brazilian Amazon
Source: Pathogens. 2020 Dec 30;10(1):19. doi: 10.3390/pathogens10010019 (PMC7823330; doi:10.3390/pathogens10010019)
Supplement: Supplementary file 1 [file pathogens-10-00019-s001.pdf]

Table S1. Oligonucleotide primers and probes used in qPCR assays.

Primer		Sequence 5′- 3′
Pv 18S	VIVAX_Fw	GCT TTG TAA TTG GAA TGA TGG GAA T
	VIVAX_Rev	ATG CGC ACA AAG TCG ATA CGA AG
	VIVAX_Probe	VIC-AGC AAC GCT TCT AGC TTA-MGB-NFQ
Pv Mt	PvMt_cox1_Fw	TTATATCCACCATTAAGTACATCACTT
	PvMt_cox1_Rv	AACCTTTAGATCTTAGATGCATTACA
	PvMt_cox1_Probe	FAM-CCTGTTGCAGTAGATGTTATCATTG-BHQ1

Gruenberg et al., 2018 [14].

Table S2. Recurrence of patients that were positive when tested using *Pv*-mtCOX1 qPCR on D28 and reported on the SIVEP-Malaria database (TBS) up to 180 days after follow-up.

Day of SIVEP-Malaria report	No. of positive samples by *Pv*-mtCOX1 qPCR on D28 n (%)	Light microscopy on D28
No notification in the system	10/29 (35)	Negative
D28	5/29 (17)	Positive
D29-D34	6/29 (21)	Negative
D35-D42	6/29 (21)	Negative
D98	1/29 (3)	Negative
D129	1/29 (3)	Negative
